# Bacterial Endophytes Isolated from Plants in Natural Oil Seep Soils with Chronic Hydrocarbon Contamination

**DOI:** 10.3389/fmicb.2016.00755

**Published:** 2016-05-24

**Authors:** Rhea Lumactud, Shu Yi Shen, Mimas Lau, Roberta Fulthorpe

**Affiliations:** Department of Physical and Environmental Sciences, University of Toronto-ScarboroughToronto, ON, Canada

**Keywords:** endophytic bacteria, hydrocarbon degradation, phytoremediation

## Abstract

The bacterial endophytic communities of four plants growing abundantly in soils highly contaminated by hydrocarbons were analyzed through culturable and culture-independent means. Given their tolerance to the high levels of petroleum contamination at our study site, we sought evidence that *Achillea millefolium, Solidago canadensis, Trifolium aureum*, and *Dactylis glomerata* support high levels of hydrocarbon degrading endophytes. A total of 190 isolates were isolated from four plant species. The isolates were identified by partial 16S rDNA sequence analysis, with class Actinobacteria as the dominant group in all species except *S. canadensis*, which was dominated by Gammaproteobacteria. *Microbacterium foliorum* and *Plantibacter flavus* were present in all the plants, with *M. foliorum* showing predominance in *D. glomerata* and both endophytic bacterial species dominated *T. aureum*. More than 50% of the isolates demonstrated degradative capabilities for octanol, toluene, naphthalene, kerosene, or motor oil based on sole carbon source growth screens involving the reduction of tetrazolium dye. *P. flavus* isolates from all the sampled plants showed growth on all the petroleum hydrocarbons (PHCs) substrates tested. Mineralization of toluene and naphthalene was confirmed using gas-chromatography. 16S based terminal restriction fragment length polymorphism analysis revealed significant differences between the endophytic bacterial communities showing them to be plant host specific at this site. To our knowledge, this is the first account of the degradation potential of bacterial endophytes in these commonly occurring pioneer plants that were not previously known as phytoremediating plants.

## Introduction

Petroleum hydrocarbons (PHCs) are the most common organic contaminants worldwide that pose human and environmental health concerns (Heiss-Blanquet et al., 2005; Canadian Council Ministers of the Environment, 2014). The proposed development of thousands of kilometers of new oil pipelines in North America forebodes more spills and leakages in the future. With expensive cleanup options, there is a high likelihood that contaminated sites will not be adequately dealt with (Commissioner of the Environment and Sustainable Development, 2012). Phytoremediation, the use of plants and their associated microorganisms, is a cost-effective and low impact remedial technology for the cleanup of contaminated soils (Pilon-Smits, 2005). Recent research reveals the high potential for optimizing phytoremediation efficiency through plant-bacterial endophyte partnerships (Barac et al., 2004; Phillips et al., 2008; Weyens et al., 2009b; Yousaf et al., 2011; Kang et al., 2012; Kukla et al., 2014). Bacterial endophytes are non-pathogenic bacteria that reside within the living tissues of the plant without harming the plant hosts (Schulz and Boyle, 2006). Some endophytic bacteria can contribute to contaminant degradation *in planta* and many are also known to promote plant growth and enhance defense mechanisms against pathogens and toxicity (Glick, 2003; Compant et al., 2010).

The successful establishment of phytoremediating plants and associated degradative bacteria in the field remains challenging. In order for phytoremediation of PHCs to be successful, both the plant and its bacterial commensals must survive in a PHC contaminated site. Inoculant strains are often rapidly out-competed by indigenous bacteria in plants (Cunningham and Ow, 1996; Afzal et al., 2012). Thus, indigenous bacteria associated with plants that are naturally growing in a contaminated site show more promise for detailed studies and future applications. A landmark study by Siciliano et al. (2001) demonstrated that selection favors bacterial endophytes that are able to degrade proximal soil contaminants or that enhance the host plant survival in the stressful environment (Germaine et al., 2009; Weyens et al., 2009a).

Most of the studies examining the potential of endophytic bacterial communities in remediating petroleum hydrocarbon from soil have been carried out in either soil with simulated petroleum contamination or in experimental pot experiments (Siciliano et al., 2001; Andria et al., 2009; Yousaf et al., 2010; Afzal et al., 2011, 2012). In this study, we characterized the bacterial endophytic communities of four plant species: *Achillea millefolium, Solidago canadensis, Trifolium aureum*, and *Dactylis glomerata*, that were growing abundantly in an oil field that has been in operation since the mid nineteenth century. The soils there experience recurring oil spills and high levels of petroleum volatiles in the atmosphere. Soil TPH concentrations at this site reach as high as 300,000 ppm (Liu, 2012). Even prior to drilling activities, natural oil seeps in the area exposed local vegetation to PHCs. These plant species, widely distributed all throughout Ontario and most provinces in Canada (Brouillet et al., 2010), have not been shown to phytoremediate petroleum hydrocarbon in soil. To our knowledge, the bacterial endophytic communities of these commonly growing plants have not been previously studied in terms of their potential in phytoremediating petroleum hydrocarbon. We hypothesize that these pioneer plants harbor diverse endophytic bacterial communities that have high petroleum hydrocarbon degrading potential. The overall goal of this study is to characterize and compare the endophytic bacterial communities and hydrocarbon degrading potential of these highly petroleum hydrocarbon tolerant plants.

## Materials and methods

### Site description and sampling

**Figure 1:**
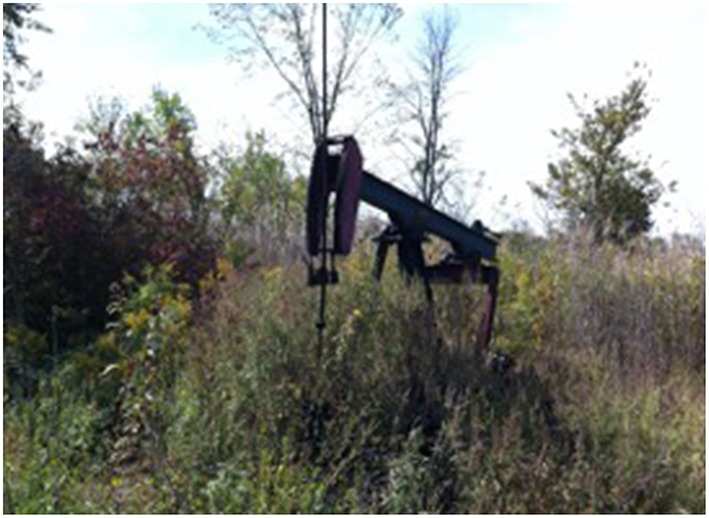
**Old-fashioned oil pumping well**. Plants were sampled around the vicinity.

The study site was located in Oilsprings, Ontario, Canada (N42°46. 267, W82°05.53864) is a natural area in which near surface oil deposits frequently seep to the top and form tar like patches. These deposits were tapped in the late 1850's and by 1861 a network of oil pumps that yielded 6000 barrels of oil per day had been established. The well systems still support an ongoing family business. The servicing of the individual pumps leads to frequent oil spillage onto the nearby soils, from which vegetation grows regardless (Figure 1).

#### Soil characterization

Bulk soils were taken from each sample site and were used to analyze total petroleum hydrocarbon (TPH), soil texture, soil pH, and total P and N. TPH concentrations were measured using TPH analyzer (INFRACAL model CVH). Soil pH was measured in a soil:water slurry (ratio of 1:2), the sedimentation method was used to determine soil texture (Gee and Bauder, 1986), % organic carbon was estimated based on loss on ignition. Total Nitrogen was determined using Dumas method (following LECO FP428 instructions/operations manual), while Olsen P was used to determine total Phosphorus (Reid, 1998).

#### Sampling

Sampling was carried out in May of 2013. Triplicate sampling pools of plants (at least five individual plants of each plant species in each sample site), of the same size and maturity from three pumping wells, from the following species: *A. millefolium, S. canadensis, T. aureum*, and *D. glomerata*, were taken from within 2 m from pumping wells with recurring oil spillage (Figure 1). Samples were stored at 4°C in zip lock bags until analyses, which were carried out within 48 h.

### Culture based analysis: endophyte isolation and identification

Stem samples, 15–20 g of each plant replicate were surface-sterilized using a series of washes (70% ethanol; 1.2% bleach with 0.1% Tween 20; followed by six washes of sterile distilled water). To test the efficacy of sterilization, an aliquot of the last washing was plated onto agar plates and sterilized stem samples were imprinted onto both Reasoner's 2A (R2A) and Tryptic Soy Agar (TSA) media. Following this, the sterilized stems were macerated in a Waring blender at 20000 rpm using sterile 60 ml 50 mM Tris-HCl and heterotrophic bacteria were isolated by plating 100 μL on R2A and TSA plates. The media plates were incubated at 28°C for a period of 1–4 weeks. Individual bacterial colonies were isolated and purified as they appeared. Lysates were made from pure colonies by boiling 2 loopfuls of 1 μL sterile disposable loops in 100 μL sterile distilled water for 7 min. One microliter of lysate was used as template in a PCR reaction using 16SrRNA primers 27F (5′-AGAGTTTGATYMTGGCTCAG-3′) and 1492R (5′-TACCTTGTTACGACTT-3′; Frank et al., 2008). The PCR reaction was as follows: 25 μL reactions with a final concentration of 0.5 mM of the forward primer and reverse primer, 1.5 mM MgCl_2_, 200 mM of each dNTP, 2.5 units of HotStarTaq *Plus* DNA polymerase (Qiagen, Canada). The PCR amplifications were carried out in a PTC-200 thermal cycler (MJ Research Inc.) with the following conditions: initial denaturing at 95°C for 5 min followed by 35 cycles of: denaturing at 95°C for 1 min, annealing at 56°C for 1 min and extension at 72°C for 1 min; final extension at 72°C for 10 min. The resultant amplicons were purified (GenElute PCR clean-up kit, Sigma Aldrich) before being sent for Sanger sequencing at The Centre for Applied Genomics (TCAG) sequencing facility (Toronto, Canada) using 27F (5′-AGAGTTTGATYMTGGCTCAG-3′) primer. The obtained sequences were subjected to BLAST analysis with the National Center for Biotechnology Information database to identify the most similar 16S rDNA sequences.

Isolates were screened for catabolic genes for enzymes: alkane hydroxylase (*AlkB*), using primers alkBwf 5′-AAYACNGCNCAYGARC TNGGVCAYAA-3′ and alkBwr 5′-GCRTGRT GRTCHGARTGNCGYTG-3′ that targets groups belonging to *Acinetobacter, Pseudomonas* and *Rhodococcus* (Wang et al., 2010). PCR conditions were initial denaturing at 94°C for 4 min followed by 32 cycles of: denaturing at 94°C for 30 s, annealing at 55°C for 30 s and extension at 72°C for 1 min; final extension at 72°C for 10 min. Catechol 2,3-dioxygenase (*C23O*) genes were assayed using primers C23O-F-AGGTGCTCGGTTTCTACCTGGCCGA and C23O-R-ACGGTCATGAATCGTTCGTTGAG (Luz et al., 2004) using PCR conditions- initial denaturing at 94°C for 4 min followed by 30 cycles of: denaturing at 94°C for 1 min, annealing at 60°C for 1 min and extension at 72°C for 1 min; final extension at 72°C for 3 min.

### Culture-independent analysis: terminal restriction fragment length polymorphism (TRFLP)

The total community DNA was extracted from plant macerates using FastDNA SPIN Kits (MP Biomedicals) following manufacturer's instructions with modifications [addition of 100 μL of protein precipitation solution (PPS) solution to the lysing solution and two additional SEW-SM washes of the bound DNA]. Community structure and taxonomic diversity were examined using TRFLP of PCR-amplified 16S rRNA gene fragments. The genomic DNA was initially amplified with universal bacterial primers 27F (5′-AGAGTTTGATYMTGGCTCAG-3′) and 1492R (5′-TACCTTGTTACGACTT-3′) using conditions as above. The resultant amplicons were digested with restriction enzymes *Pvu*II and *Msc*I (NEB Canada) to minimize chloroplast interference (Shen and Fulthorpe, 2015). One microliter of the resultant digested product was used as template in PCR reaction (same conditions as above) using 16S rRNA fluorescein labeled primers, 27F-FAM and 1492R-HEX (LifeTechnologies, Canada). The generated amplicons were digested with restriction enzyme *Msp*I and sent to the Agriculture and Food laboratory at the University of Guelph for fragment analysis.

### Community composition comparisons

TRFLP data (terminal 16S fragments representing phylotypes present in the plant tissues) were used to determine phylotype densities and richness. For the T-RFLP data, fragments < 60 bp in size were omitted in the analysis. The Microsoft Excel macro Treeflap (Rees et al., 2005), obtained from http://urbanstreams.net/index.php/the-treeflap-macro/, was used to round the fragment sizes to the nearest one base pair and to align the fragments of the same size from different samples, generating a cohesive table that contained the different fragments sizes and their relative heights in each sample. The height data was converted to % abundance and any fragments that had < 1% abundance were omitted. This T-RFLP data set was subjected to Non-metric Multidimensional Scaling (NMDS) based on Bray Curtis dissimilarities and Adonis tests, using programs of the vegan package (Oksanen et al., 2012) for R version 2.15.2 (R Core Team, 2012).

Analysis of Variance tests (ANOVAs) were used to compare plant species with respect to total culturable heterotrophic bacteria and phylotype richness, thereafter; a *post-hoc* test was done using bonferroni correction. Tests of homoscedacity were carried out prior to performing ANOVAs. All these were done using Microsoft excel.

### Screening for petroleum hydrocarbon degrading potential of bacterial endophytes

Mineralization of PHC by individual endophyte isolates was evaluated by screening for respiration activity in microplate assays, using Bushnell-Haas medium amended with 2% of motor oil or kerosene, or 1 mM concentrations of toluene, octanol or naphthalene. Filter-sterilized *p*-iodonitrotetrazolium violet concentration was added onto each well at 1 mM, isolates that were putative oil-degraders reduced the dye to purple. For positive controls, Bushnell-Haas medium with glycerol and Pseudomonas F broth, inoculated with any endophytic bacteria in the culture collection was used. Bushnell-Haas medium amended with petroleum derivatives mentioned above with sterile water was used as negative control.

### Analysis using GC-FID

The PHC degradation capability of the isolates that strongly exhibited (showing dark purple) degrading potential in colorimetric assays were further tested by inoculating them in Bushnell-Haas minimal medium amended with 1 mM concentrations of toluene or naphthalene using PTFE-lined screw capped tubes. The inoculated tubes were placed in incubated shaker at 27°C. After 1 week of incubation, residual hydrocarbons were extracted using hexane as extraction solvent following a method modified from Michaud et al. (2004). Toluene and naphthalene concentrations were determined using a Gas Chromatography-Flame Ionization Detector (Agilent Technologies 7890A GC System; using HP-5 30 m × 0.32 mm × 0.25 um column, helium carrier gas, hexane solvent) and compared to concentrations in uninoculated controls incubated and shaken for the same period of time.

## Results

### Soil characterization

TPH concentrations from soil sediment immediately soaked in oil from the pumping leakage ranged from 250,000 to 300,000 ppm, while the soils surrounding the wells where the plants are growing had a concentration range of 45,000–50,000 ppm. The site has silty clay loam soil texture, with pH of 6.8–7.3, organic content of surrounding unoiled soils ranges from 20 to 30% organic carbon, extractable total P and total N were 21 mg/L and 0.44% dry soil, respectively.

### Culturable endophytic bacterial community characterization

The number of culturable endophytic bacteria recovered was highest in *D. glomerata* (mean of 531 colony-forming units (cfu) per gram fresh weight), while *T. aureum* had the lowest number of isolates recovered with 150 cfu/g (Table 1). Endophytic bacterial population densities were significantly different between *D. glomerata* and *T. aureum* (*P* < 0.05). Conversely, phylotype richness, as determined by the number of distinguishable culture-independent TRFLP fragments ranged from 18 to 23, which was not significantly different among plant species (*P* < 0.05), with *S. canadensis* and *A. millefolium* having the lowest and highest phylotype richness, respectively.

**Table 1:** **Mean values (*N* = 3) of colony forming units and species richness using TRFLP fragments of endophytic bacterial communities per gram fresh weight of plant tissues recovered from *Achillea millefolium, Dactylis glomerata, Trifolium aureum*, and *Solidago canadensis*, standard deviation in parenthesis**.

**Plant**	**CFU/g**	**Phylotype richness (TRFLP fragments)**
*Achillea millefolium*	489(117)^ab^	22(6)^a^
*Dactylis glomerata*	531(75)^a^	23(4)^a^
*Solidago canadensis*	353(96)^ab^	18(2)^a^
*Trifolium aureum*	150(72)^b^	22(3)^a^

*Means with different letters are significantly different at 5% level of confidence*.

**Figure 2:**
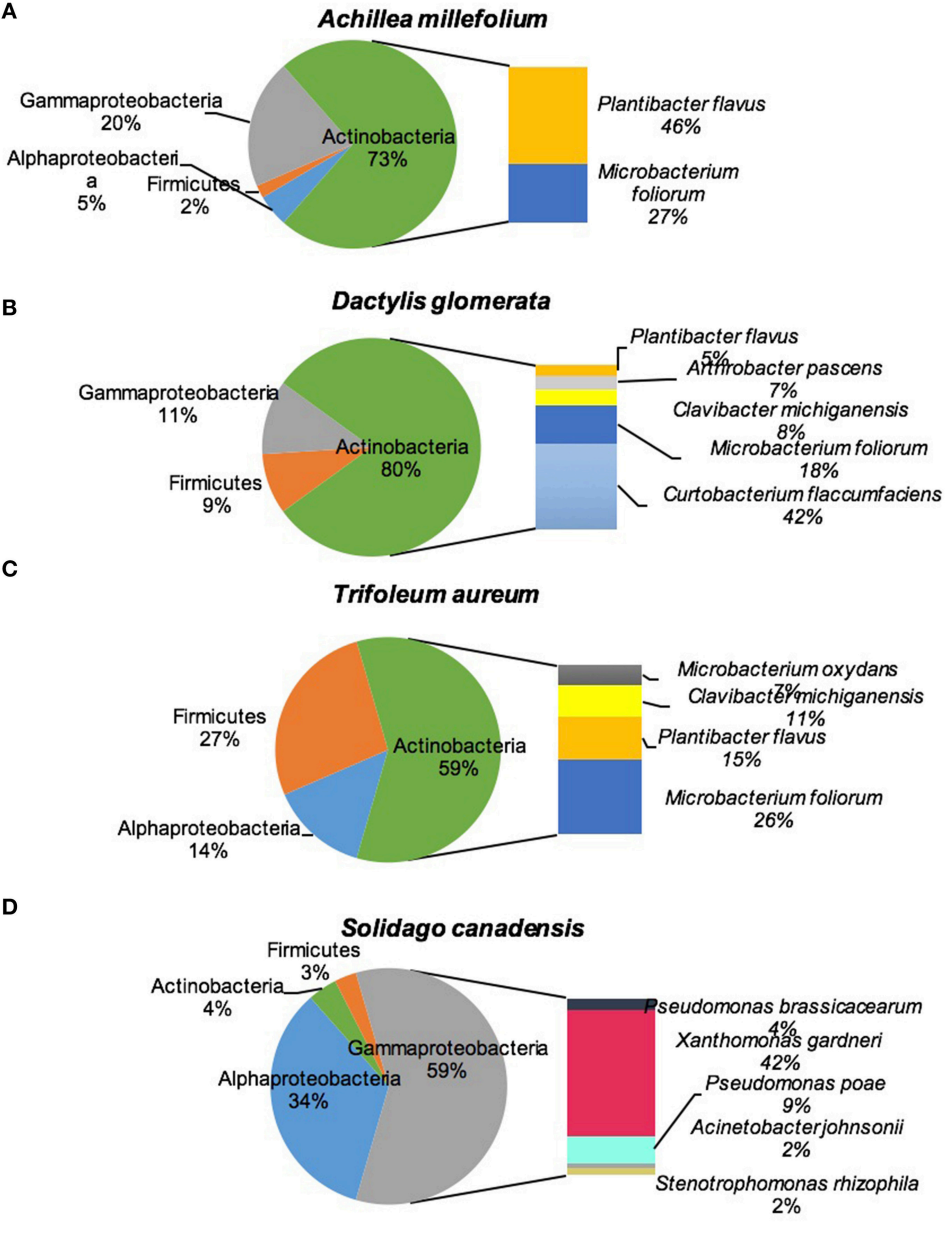
**Diversity of cultivable bacteria isolated from stems of four plant species: (A) *Achillea millefolium*, (B) *Dactylis glomerata*, (C) *Trifolium aureum*, (D) *Solidago canadensis***.

Figure 2 shows the culturable endophytic community composition expressed as percentage abundance of isolates found in each represented phylum. All the plant species were dominated by class Actinobacteria except for *S. canadensis* which was dominated by Gammaproteobacteria. Around half (46%) of the total isolates in *A. millefolium* were *Plantibacter flavus*, 27% were identified as *Microbacterium foliorum*, and 10% came from *Stenotrophomonas rhizophila*. *M. foliorum* and *P. flavus* were present in all the plants, with *M. foliorum* showing predominance in *D. glomerata* and both species dominated *T. aureum* (Figure 2). *D. glomerata* was also dominated by *Curtobacterium flaccumfaciens* (42%) and *Pseudomonas poae* (11%). The bacterial endophytes that were >10% in *T. aureum* were *P. flavus* (15%), *Clavibacter michiganensis* (11%), and *Rhizobium* sp. (10%). *S. canadensis* plant was dominated by *Xanthomonas gardneri* (42%), *Rhizobium* sp. (32%), and *Pseudomonas poae* (9%; Figure 2).

### Mineralization assay

Table 2 shows PHC degrading capabilities of this endophytic bacterial collection. The PHC degradation potential of the recovered isolates was assessed through their ability to reduce tetrazolium dye when provided with kerosene, octanol, toluene, naphthalene, or motor oil as sole carbon sources. More than 70% of the isolates in each of the plant species show putative PHC degradation according to this test. All the culturable predominant taxa have petroleum hydrocarbon degradation potential (Table 2). *M. foliorum*, which was present in all plant species, showed differing PHC oxidation capabilities depending on their source. *M. foliorum* from *A. millefolium* and *T. aureum* plants did not show oxidation of octanol and toluene, respectively, whereas, *M. foliorum* from both *D. glomerata* and *S. canadensis* were able to oxidize all the PHC compounds. Conversely, *P. flavus*, another species that was isolated from all plants, showed oxidation of all the PHC substrates. *A. millefolium* showed the highest proportion of culturable EBs with PHC degradation potential (91%), followed by *S. canadensis* (80%), *T. aureum* (75%), and *D. glomerata* (74%). Across all plants, EB communities showed highest potential (67–87%) for degrading naphthalene, while lowest on motor oil (28–46%; Table 2).

**Table 2:** **Hydrocarbon degrading potential (through colorimetric mineralization assay) of dominant cultivable endophytic bacteria isolated from different plant species**.

**Plant**	**% degraders**	**Dominant taxa (closest relative)**	**CFU/g tissue**	**No. of isolates assayed**	**Kerosene**	**Octanol**	**Toluene**	**Naphthalene**	**Motor oil**
*Achillea millefolium* (54 isolates)	91^a^				63%^a^	57%^a^	67%^a^	87%^a^	46%^a^
		*Microbacterium foliorum*	132	8	+	−	+	+	+
		*Plantibacter flavus*	225	14	+	+	+	+	+
		*Stenotrophomonas rhizophila*	48	6	+	+	+	+	+
*Dactylis glomerata* (85 isolates)	74^a^				59%^a^	48%^a^	57%^a^	67%^a^	39%^a^
		*Arthrobacter pascens*	37	9	+	+	+	+	+
		*Curtobacterium flaccumflaciens*	223	12	+	+	+	+	−
		*Microbacterium foliorum*	95	8	+	+	+	+	+
		*Plantibacter flavus*	27	4	+	+	+	+	+
		*Pseudomonas poae*	58	4	+	+	+	+	−
*Trifolium aureum* (12 isolates)	75^a^				50%^a^	42%^a^	42%^a^	67%^a^	42%^a^
		*Clavibacter michiganensis*	17	2	−	−	−	+	−
		*Microbacterium foliorum*	39		+	+	−	+	+
		*Microbacterium oxydans*	11	1	+	+	+	+	+
		*Plantibacter flavus*	23	1	+	+	+	+	+
		*Rhizobium*	14	1	+	+	+	+	+
*Solidago canadensis* (39 isolates)	80^a^				67%^a^	46%^a^	72%^a^	74%^a^	28%^a^
		*Pseudomonas poae*	32	3	+	+	+	+	−
		*Rhizobium*	106	5	+	+	−	+	+
		*Xanthomonas gardneri*	148	7	+	−	+	+	+

a *Percentage of isolates from total isolates assayed in each plant species that showed positive in colorimetric assay*.

The isolates that showed the most reduction of the tetrazolium dye in microplate assays were tested further for their PHC degradation potential using toluene (tol) and naphthalene (nah), which were analyzed using GC-FID technique. Mineralization rates of the predominant strains were shown in Table 2. The number of isolates that were tested and number that showed ≥20% degradation in each plant host were: *A. millefolium*-14, tol/nah degraders-9 (64%); *D. glomerata*-56, tol/nah degraders-23 (41%); *T. aureum*-7, tol/nah degraders-6 (86%); *S. canadensis*-23, tol/nah degraders-8 (35%). A detailed list showing endophytic bacterial strains in each plant host that demonstrated ≥20% mineralization is presented in Table 3. All isolates were also screened for presence of *alkB* gene, which showed to be negative in all the isolates except for *P. poae*; and negative for *C23O* gene in all the isolates.

**Table 3:** **List of taxa showing ≥20% mineralization in each plant host species**.

**Plant source**	**Putative ID**	% mineralization (GC-FID)^*^	
		**Toluene**	**Naphthalene**
*Achillea millefolium*	*Agrobacterium tumefaciens* 255	34	36
(Total = 14^a^)	*Agrobacterium tumefaciens* 274	34	36
	*Bacillus* sp.	34	31
	*Microbacterium foliorum*	34	36
	*Plantibacter flavus* 251	37	37
	*Plantibacter flavus* 258	38	36
	*Plantibacter flavus* 259	53	39
	*Plantibacter flavus* 279	53	37
	*Stenotrophomonas rhizophila*	33	41
*Dactylis glomerata*			
(Total = 56^a^)	*Aeromicrobium* sp.	29	−
	*Arthrobacter defluvii*	13	28
	*Bacillus aryabhattai* 143	23	−
	*Bacillus aryabhattai* 159	94	20
	*Chryseobacterium* sp. 127	53	41
	*Chryseobacterium* sp. 128	30	41
	*Clavibacter michiganensis*	53	40
	*Curtobacterium flaccumflaciens* 153	21	13
	*Curtobacterium flaccumflaciens* 155	9	33
	*Curtobacterium flaccumflaciens* 185	23	20
	*Curtobacterium flaccumflaciens* 200	31	32
	*Curtobacterium* 198	13	32
	*Curtobacterium herbarum*	23	−
	*Methylobacterium brachiatum*	24	−
	*Microbacterium foliorum* 117	53	41
	*Microbacterium foliorum* 122	17	20
	*Microbacterium foliorum* 139	11	26
	*Mycobacterium aubagnese*	−	22
	*Plantibacter flavus* 126	16	24
	*Plantibacter flavus* 140	29	16
	Iso 201 (not sequenced)	−	20
*Solidago canadensis*			
(Total = 23^a^)	*Brevundimonas nasdae* 210	53	39
	*Brevundimonas nasdae* 211	35	41
	*Microbacterium oleivorans*	48	32
	*Pseudomonas brassicacearum* 205	33	31
	*Pseudomonas brassicacearum* 206	30	34
	*Xanthomonas gardneri* 209	49	40
	*Xanthomonas gardneri* 212	20	35
*Trifoleum aureum*			
(Total = 7^a^)	*Clavibacter michiganensis*	52	41
	*Microbacterium foliorum*	51	37
	*Microbacterium oxydans*	36	41
	*Plantibacter flavus*	37	23
	*Rhizobium* sp.	32	36
	Iso 234	28	35

a *Total number of isolates tested*.

* *corrected from HC loss from negative control tubes*.

– *no loss*.

### Culture-independent analysis of bacterial endophytic community

**Figure 3:**
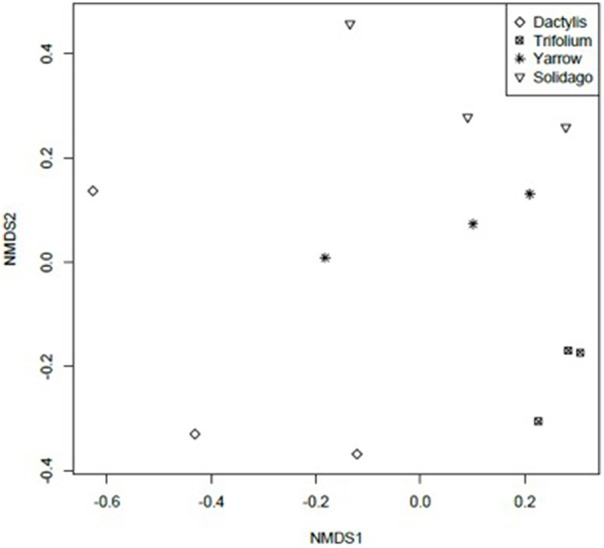
**Non-Metric Multidimensional Scaling of TRFLP profiles of bacterial endophytic communities from different plant species**. Stress: 0.07. *Achillea millefolium* (Yarrow), *Dactylis glomerata* (Dactylis), *Trifolium aureum* (Trifolium), *Solidago canadensis* (Solidago).

Culture-independent analysis of endophytic bacterial communities was done using TRFLP fragments. NMDS analysis of TRFLP fragments shows that endophytic bacterial community structures among plant species tended to be plant species specific (Figure 3). TRFLP fragments were subjected to permutation multivariate analysis of variance using Adonis function, results revealed significant differences (*P* < 0.05, F model = 2.40, *R*^2^ = 0.47) of endophytic bacterial phylotypes of differing plant species.

As in culturable endophytic bacterial species, there were 4 phylotypes common to all plant species with sizes: 115, 119, 487, and 491 bp. There were 2 phylotypes shared by *T. aureum, A. millefolium*, and *S. canadensis*: 128 and 337 bp. The plants *A. millefolium* and *S. canadensis* both belonging to family Asteraceae shared phylotypes: 219, 401, and 402 bp. None of these fragment lengths correspond to the dominant culturable strains however.

## Discussion

PHCs are known to be toxic to plants, 10,000 ppm of crude oil inhibits the growth of corn and red beans (Baek et al., 2004). Chaîneau et al. tested phytotoxicity of fuel oil hydrocarbons on sunflower, maize, barley, wheat, bean, clover and lettuce, and the plants responded in a species specific manner—showing toxicity (reduced plant growth and inhibition of seed germination) at concentrations ranging from 3000 to 12000 ppm (Chaîneau et al., 1997). Crude oil concentrations ranging from 40,000 to 60,000 ppm were also found to have adverse effects on germination and plant growth of forest fruit tree species *Dacryodes edulis* in Nigeria (Agbogidi and Eshegbeyi, 2006). Despite the reported phytotoxic effects of PHCs, common herbaceous plants, such as, *A. millefolium, S. canadensis, T. aureum*, and *D. glomerata*, were abundantly growing in soils near oil pumping wells that occasionally spills crude oil at TPH concentrations reaching to as high as ~300,000 ppm. Thus, we hypothesize that these high concentration likely selected for historical plants with endophytic bacteria with the capacity to degrade PHCs. In this study, we present the community structure and petroleum hydrocarbon degradation potential of bacterial endophytes isolated from commonly growing plants that are relatively understudied in phytoremediation.

Cultivable endophytic bacterial population densities from the stems of these plants were lower compared to bacterial endophytes from agricultural crops that were three to four magnitudes higher (Hallmann et al., 1997). *D. glomerata*, having the highest population density, belongs to family Poaceae, which has a dense, fibrous root system that enable plants to penetrate the soil on a wider span laterally providing a large surface area (Kaimi et al., 2006). As endophytes are widely believed to be derived from rhizopheric bacterial community, we assume the greater root surface would expose it to more colonizers.

Culturable endophytic Actinobacteria were common in our study plants. Although the species *M. foliorum* in particular has not been reported as a hydrocarbon degrader, other members of the genus certainly have been, such as, *Microbacterium oleivorans* and *Microbacterium hydrocarbonoxydans* (Schippers et al., 2005; Ali et al., 2012; Kukla et al., 2014). A *P. flavus* is not known to be petroleum hydrocarbon degraders, nor are any members of this genus. The group Actinobacteria was found to be predominant in the stems of a leguminous plant *Cystisus striatus* (Broom) growing in hexachlorocyclohexane-contaminated soil (Becerra-Castro et al., 2011), in the bacterial endophytic communities isolated from poplar (Ulrich et al., 2008), *A. thaliana* roots (Bulgarelli et al., 2012) and in both the culture collection and clone libraries isolated from potato. The latter environment was dominated by *Arthrobacter* sp. and *Microbacterium* sp. (Someya et al., 2013).

More than 50% of the culture collection isolated from all the sampled plants have the ability to degrade at least one of the PHCs (kerosene, octanol, toluene, naphthalene, and motor oil) that were tested. The majority of the isolates from all the plants (67–87%) demonstrated putative degradation of naphthalene (Table 2). Naphthalene, a natural component of crude oil, is a low molecular polyaromatic hydrocarbon that has lower volatilization from the soil than short chain alkanes and monoaromatics; and also less likely to bind to soil organic matter than higher molecular weight alkanes and polyaromatics, and therefore may partition more into plant tissues than the other compounds. Naphthalene is not only translocated from the soil through the roots but is also assimilated by plants through the leaves (Fismes et al., 2002; Wang et al., 2012). Since it is easily dissipated to plants compared to higher molecular weight PAHs, naphthalene is toxic to plants, causing inhibition of growth, reduced transpiration rates, wilting, chlorosis, and plant death at concentrations 0.3–9% that varies with plant species (Henner et al., 1999; Kuiper et al., 2001; Germaine et al., 2009).

*P. flavus*, the species that was shared by all the plants that dominated in all plants except for *S. canadensis*, was able to degrade all the PHCs being tested, its degradative capabilities were similar in all the plants. Conversely, *M. foliorum*, which was predominant in all the plants had varying oxidation patterns. This may be because different strains carry different sets of genes, which was likely influenced by plant host species. The apparent absence of a*lkB* genes in most of the isolates and *C23O* genes in all the isolates suggests that divergent but isofunctional genes were responsible for alkane degradation. The a*lkB* primer we used is redundant and able to target conserved regions of the alkane monooxygenase gene found in a wide range of bacteria- *Pseudomonas, Rhodococcus*, and *Acinetobacter* groups (Wang et al., 2010). These results highlight the likelihood that we have isolated novel degraders.

To the best of our knowledge, only the endophytic *P. poae* was previously reported to be isolated from plants in a petroleum contaminated site (Phillips et al., 2008). Most of the species presented in Table 2 are new plant-associated bacteria in relation to petroleum hydrocarbon degradation. Endophytic *P. flavus* was found to be associated with a hyperaccumulator plant *Thlaspi goesingense* by Idris et al. (2004). Nickel is found in crude oil and plant enzyme urease; thus, the resistance of *P. flavus* to nickel is not surprising. *Microbacterium phyllosphaerae*, a close relative of *M. foliorum*, in a study by Salmerón-Alcocer (Salmerón-Alcocer et al., 2007), together with *Burkholderia* sp. and *Candida tropicales* formed a microbial consortium to degrade chlorophenols. *S. rhizophila* was found to be predominant in both *A. millefolium* and *S. canadensis*. In the area of bioremediation, *S. rhizophila* was reportedly isolated from the turbine-oil degrading microbial consortium (Hosokawa et al., 2010) and diesel contaminated antartic soils (Vázquez et al., 2013). A close relative, *S. maltophilia*, was found to degrade RDX (Binks et al., 1995).

NMDS TRFLP data showed that endophytic bacterial communities were host-species specific. Despite petroleum hydrocarbon exposure, which is a strong selection pressure, plant species remained a driving factor to endophytic bacterial communities. Similar results (from field experiments) was also reported on prairie plants: *Medicago sativa, Lolium perenne, Elymus angustus, Puccinellia nuttalliana, and Agropyron elongatum*, growing in weathered-hydrocarbon contaminated soil, which showed distinct endophytic microbial communities with high hydrocarbon degradation potential (Phillips et al., 2008). Bacterial endophytic communities from salt marsh plant species *Halimione portulacoides* and *Sarcocornia perennis*, commonly growing in the estuarine system which was contaminated with petroleum hydrocarbon, also showed plant species specificity (Oliveira et al., 2014). Although our results show plant-host specificity, we also show phylotypes that are common in all the plants and plants belonging to the same family, as in this case *A. millefolium* and *S. canadensis*, both belonging to family Asteraceae.

This study presented a possible rich reservoir of versatile EB degraders that is promising for the study of PHC phytoremediation mechanisms. These plants and their dominant culturable EBs with high PHC degradative potential have not been reported before in phytoremediation studies. The plants in this study are widespread in North American disturbed habitats and other temperate systems. They abundantly grow in recolonizing systems and in various soil types (Brouillet et al., 2010). These characteristics make them good candidates for phytoremediation of shallow soils. Furthermore, many of these genera were reported to have plant beneficial characteristics. Members of the group Actinobacteria are known to produce plant-growth promoters and enhance the ability of plants to withstand environmental stress (Luz et al., 2004). The genus *Stenotrophomonas* is a well-studied genus of family Xanthomonadaceae in plant-growth promotion and plant health (Ryan et al., 2009). *S. rhizophila* was reported to have antifungal properties against plant pathogenic fungi (Wolf, 2002). Plant growth promoting and contaminant-degrading bacterial endophytes are more effective in phytoremediation of polluted soils (Kukla et al., 2014). Some of these bacterial endophytes likely possess both plant growth promoting and degradative abilities, which is what we are currently investigating in the lab.

## Author contributions

RL–wrote the manuscript, implemented lab and field work, analyzed data. SS–helped in lab and field work. ML–helped in degradation experiment. RF–supervisor/PI, co-wrote the manuscript, advised lab/field work and data analyses.

### Conflict of interest statement

The authors declare that the research was conducted in the absence of any commercial or financial relationships that could be construed as a potential conflict of interest.
